# Exploring the potential mechanism and molecular targets of Taohong Siwu Decoction against deep vein thrombosis based on network pharmacology and analysis docking

**DOI:** 10.1097/MD.0000000000036220

**Published:** 2024-01-12

**Authors:** Wei Fan, Jinhui Liu, Qingyan Liu

**Affiliations:** a Department of Orthopaedics, The Affiliated Hospital of Southwest Medical University, Luzhou, Sichuan, China; b Sichuan Provincial Laboratory of Orthopaedic Engineering, Luzhou, Sichuan, China; c The Operating Room, The Affiliated Hospital of Southwest Medical University, Luzhou, Sichuan, China.

**Keywords:** deep vein thrombosis, molecular docking, network pharmacology, Taohong Siwu Decoction

## Abstract

This study aims to investigate the mechanism of Taohong Siwu Decoction (THSWD) against deep vein thrombosis (DVT) using network pharmacology and molecular docking technology. We used the Traditional Chinese Medicine Systems Pharmacology database and reviewed literature to identify the main chemical components of THSWD. To find targets for DVT, we consulted GeneCards, Therapeutic Target Database, and PharmGKB databases. We used Cytoscape 3.8.2 software to construct herb-disease-gene-target networks. Additionally, we integrated drug targets and disease targets on the STRING platform to create a protein–protein interaction network. Then, we conducted Kyoto Encyclopedia of Genes and Genomes and gene ontology analysis. Finally, We employed the molecular docking method to validate our findings. We identified 56 potential targets associated with DVT and found 61 effective components. beta-sitosterol, quercetin, and kaempferol were the most prominent among these components. Our analysis of the protein–protein interaction network revealed that IL6, L1B, and AKT1 had the highest degree of association. Gene ontology analysis showed that THSWD treatment for DVT may involve response to inorganic substances, negative regulation of cell differentiation, plasma membrane protein complex, positive regulation of phosphorylation, and signaling receptor regulator activity. Kyoto Encyclopedia of Genes and Genomes analysis indicated that lipid and atherosclerosis, pathways in cancer, as well as the PI3K-Akt pathway are the main signal pathways involved. Molecular docking results demonstrated strong binding affinity between beta-sitosterol, quercetin, kaempferol, and AKT1 proteins as well as IL1B and IL6 proteins. The main targets for THSWD treatment of DVT may include AKT1, IL1B, and IL6. Beta-sitosterol, quercetin, and kaempferol may be the active ingredients responsible for producing this effect. These compounds may slow down the progression of DVT by regulating the inflammatory response through the PI3K/Akt pathway.

## 1. Introduction

Deep vein thrombosis (DVT) is a condition where blood clots form in the deep veins due to factors like vein wall injury and stagnant blood flow. This can block or narrow the vein, leading to serious complications.^[1,2]^ DVT is highly prevalent, with studies showing that patients with femoral fractures have a preoperative incidence rate of 26.4% even with anticoagulants and physical prevention measures,^[3]^ while those undergoing major orthopedic surgery have an incidence rate of 40% to 60%.^[4]^ The danger of DVT lies in its potential to cause fatal pulmonary embolism when an embolus dislodges, resulting in high mortality rates and contributing significantly to perioperative and accidental hospital deaths.^[2,5]^ Additionally, thrombosis delays postoperative functional exercise for fracture patients, contradicting the need for early functional exercise after orthopedic surgery. Currently, low molecular weight heparin and vitamin K antagonists are commonly used in clinical practice to prevent DVT.^[6]^ However, long-term observation has revealed adverse reactions associated with their widespread use. The main reactions observed were hematoma formation, bleeding, and decreased hemoglobin concentration.^[7,8]^ However, There is ongoing controversy regarding the use of anticoagulation therapy in patients with traumatic stress ulcers, hematological disorders, or cerebral hemorrhage.^[9–11]^ Consequently, effectively preventing DVT in long-term bedridden patients without causing new complications remains an urgent issue that requires attention.

Chinese herbal medicine has been used for over a thousand years to treat and prevent DVT.^[12]^ In traditional Chinese medicine (TCM), DVT is classified as “pulse obstruction” and “blood stasis.^[13]^” Sun Simiao, a renowned Chinese physician from over a thousand years ago, stated in Qian Jin Bei Ji Yao Fang that thrombosis is primarily caused by poor blood circulation: “If qi and blood are stagnant, there will be pain; if the pulse is blocked, there will be swelling; if stagnation persists for a long time, heat will arise.” Taohong Siwu decoction (THSWD), an improved formula of Siwu decoction, is a mixture of 6 Chinese medicine extracts including Persicae Semen (Taoren, TR, the dried ripe seed of Prunus persic.), Carthami Flos (Honghua, HH, the dried Carthamus tinctorius.), Angelica sinensis radix (Danggui, DG, the dried root of Angelica sinensis), Chuanxiong Rhizoma (Chuanxiong, CX, the dried rhizome of Ligusticum chuanxiong Hort.), Paeoniae Radix Alba (Baishao, BS, the dried root of Paeonia lactiflora Pall.), Rehmanniae Radix Praeparata (Shudihuang, SDH, the dried root of Rehmannia glutinosa Libosch.).^[14]^ THSWD is a well-known and classic prescription of TCM, derived from the “Yizong Jinjian” of the Qing Dynasty. The primary focus of this prescription is to remove blood stasis, nourish blood, promote circulation, resolve stasis, regulate meridians, and benefit qi. In China, THSWD has been used for a long time to treat deep vein thrombosis (DVT). Clinical control studies have shown that THSWD effectively improves hypercoagulability in patients with intertrochanteric fracture of the femur, joint replacement, and tibiofibular fracture by reducing the incidence of DVT in lower limbs.^[15–17]^ However, current research on THSWD is limited to TCM experiences and clinical observations passed down through generations; there is little research on its molecular mechanisms.

TCM relies on complex formulas of multiple herbs, which are based on ancient texts and empirical knowledge rather than modern scientific research. This lack of quantitative evidence makes it difficult to evaluate the therapeutic effects of TCM.^[18]^ As a result, some Western scholars view it as an empirical practice without a theoretical foundation, rather than a science. These factors pose significant challenges for TCM research.^[19]^

Network pharmacology utilizes virtual computing and database retrieval methods to investigate the mechanisms of diseases and drug effects within a broader biological network context. This approach offers novel insights into the process of drug discovery.^[20]^ Its primary objective is to tackle scientific challenges at various levels, offering a promising solution to overcome limitations such as inadequate basic research in traditional Chinese medicine.^[21,22]^ In this study, we utilized network pharmacology to create target networks and protein–protein interaction (PPI) networks for drugs and diseases. We then conducted gene ontology (GO) and Kyoto Encyclopedia of Genes and Genomes (KEGG) analysis to identify biological functions and major signaling pathways. To validate our findings, we employed molecular docking technology to confirm the proposed functional components and main targets. Overall, our research combines network pharmacology with molecular docking techniques to investigate the effects of THSWD on DVT and its underlying molecular mechanisms.

## 2. Materials and methods

Ethical approval was waived or not necessary, all procedures performed in studies do not involve human participants or animals.

### 2.1. Screening of the active ingredients in THSWD

We utilized the Traditional Chinese Medicine Systems Pharmacology Database (TCMSP) platform (http://tcmspw.com/tcmsp.php) to identify the active components of THSWD (accessed in June 2023). Our selection criteria were based on absorption, distribution, metabolism and excretion (ADME) processes. Oral bioavailability (OB) is a crucial pharmacokinetic parameter in ADME, while drug-likeness is a qualitative concept used in drug design to estimate molecular characteristics of drugs.^[23]^ To identify suitable active compounds, we screened for ingredients with OB ≥ 30% and drug-likeness ≥ 0.18. This ensured that the selected compounds possessed both desirable OB and drug-like properties.^[24,25]^

### 2.2. Construction of drug active ingredient target network and identification of DVT predictive targets

After identifying the effective target of THSWD in the TCMSP database, we standardized the conversion of target symbols and gene symbols using the UniProt database (https://www.uniprot.org/, accessed in June 2023). To obtain disease targets from various databases, we searched for “deep venous thrombosis” as a keyword in GeneCards (https://www.genecards.org/, relevance score ≥ 2.0, accessed in June 2023), Therapeutic Target Database (https://db.idrblab.net/ttd/, accessed in June 2023), and PharmGKB (https://www.pharmgkb.org/, accessed in June 2023) databases.^[26–28]^ We then merged these targets, removed duplicates, and collected remaining DVT targets. Using Cytoscape network visualization software version 3.8.2, we constructed a “herb-disease-gene-target” network of effective ingredients and action targets. Finally, we analyzed network characteristics to elucidate interactions between effective ingredients and targets in herbs and diseases using plug-ins available within this software.

### 2.3. Construction and analysis of protein-protein interaction (PPI) network

The STRING (Search Tool for Tetrieving Interacting Genes/proteins) database (https://string-db.org/, accessed in June 2023) was utilized to predict protein–protein interactions. Overlapping targets were introduced to create the PPI network.^[29]^ Subsequently, the PPI file was imported into Cytoscape 3.8.2 for constructing a core PPI network diagram.

### 2.4. GO enrichment analysis and KEGG pathway analysis

To better understand the biological functions and signaling pathways associated with DVT, we utilized the Metascape database (https://metascape.org/, accessed in June 2023)^[30]^ to conduct GO and KEGG enrichment analyses. The GO analysis helped us identify relevant biological processes (BP), cellular components, and molecular functions. Meanwhile, the KEGG enrichment analysis allowed us to pinpoint significant signaling pathways involved in these BP. We set a *P* value cutoff of *P* < .01, where a smaller *P* value indicates a higher likelihood of an authentic enrichment outcome rather than a random occurrence. In this study, we used −log 10 (*P* value) as our unit of measurement; as this value increases, so does the reliability of the enrichment result.

### 2.5. Molecular docking

Molecular docking is a simulation method used in drug design to predict how molecules interact with receptors and ligands.^[31]^ Autodock Tools is software that helps researchers understand the interaction between protein targets and small molecules.^[32]^ In this study, we used molecular docking to investigate whether the core components of THSWD identified through network pharmacology could bind with core proteins. We selected the top 3 compounds based on degree value from the “herb-disease-gene-target” network’s core components of THSWD, and chose the top 3 proteins based on degree value from PPI network’s core targets. Corresponding 3D structure files for proteins and small molecule compounds were downloaded from RCSB database (https://www.rcsb.org/ accessed in June 2023) and TCMSP database,^[33]^ dehydrated and hydrogenated before importing them into Autodock Tools (ver.1.5.6). We used AutoDock Tools software to perform molecular docking on selected proteins and small molecules. Prior to docking, we modified, dehydrated, and hydrogenated the molecules. To generate binding mode diagrams, we imported the docked protein and small molecule files into PyMOL (version 1.8.x). By using the “find-polar contacts-to any atom” option in PyMOL, we were able to automatically identify hydrogen bonds or other interactions.^[34]^

## 3. Results

### 3.1. Screening of effective components in THSWD

According to the established screening criteria, we have identified a total of 61 THSWD active ingredients (see Table S1, Supplemental Digital Content, http://links.lww.com/MD/L267).

### 3.2. Compound-target network and analysis

**Figure 1.**
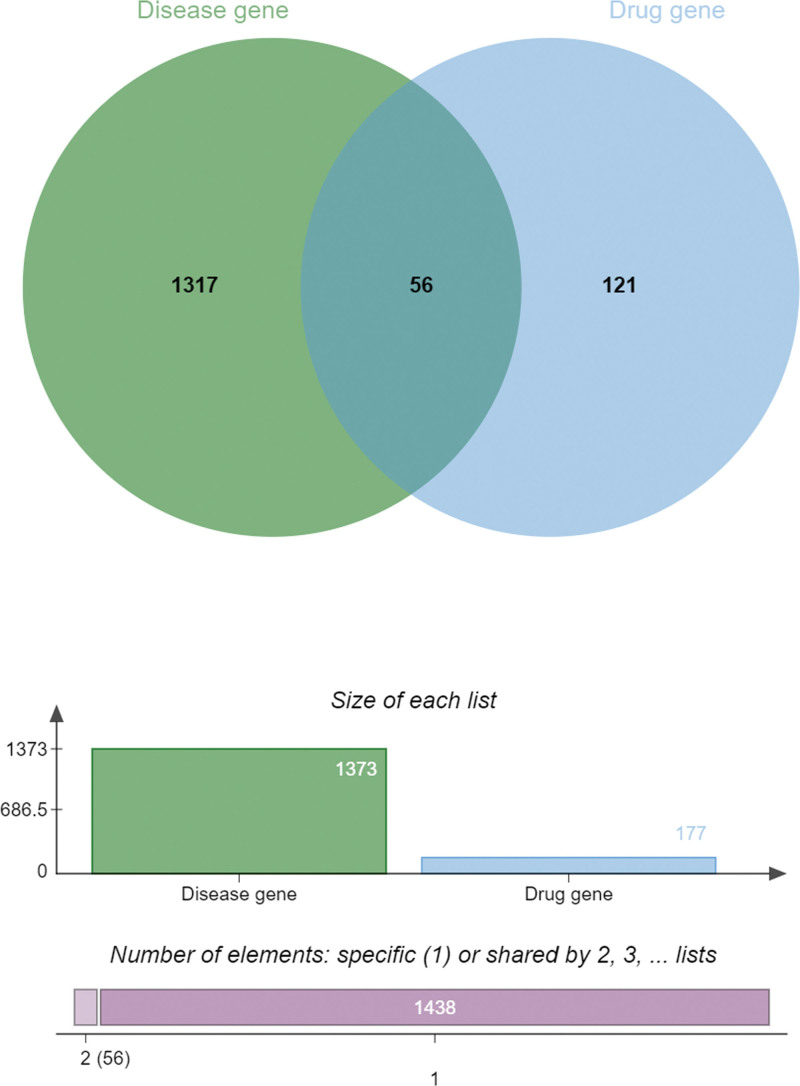
Venn diagram showing the overlapping target genes for THSWD against DVT. DVT = deep vein thrombosis; THSWD = Taohong Siwu Decoction.

**Figure 2.**
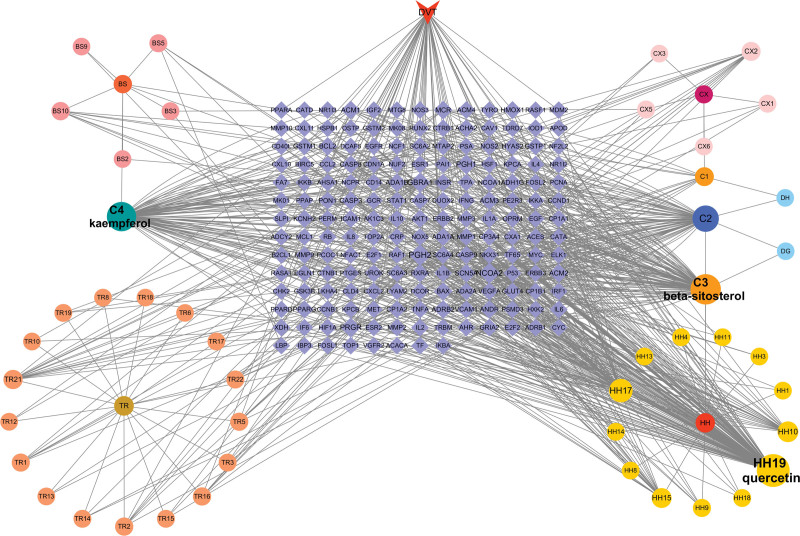
Herb-disease-gene-target network of THSWD against DVT. The larger the font size, the more important its role in the compound. THSWD, Taohong Siwu Decoction; DVT, Deep vein thrombosis; TR, Tao Ren; HH, Hong Hua; DG, Dang Gui; CX, Chuan Xiong; BS, Bai Shao; DH, Di Huang; C1, Sitosterol, common components of DH, BS and CX; C2, Stigmasterol, common components of DH, HH and DG; C3, Beta-sitosterol, common components of TR, HH, DG and BS; C4, Kaempferol, common components of BS and HH.

We conducted a search in the Therapeutic Target Database, PharmGKB, and GeneCards databases to identify potential targets related to DVT. After removing duplicate targets, we obtained a list of 1373 potential targets. Additionally, we organized the results from TCMSP, eliminated duplicate sites, and matched drug targets with disease targets. This process resulted in 56 core targets (Fig. 1). To further explore the interaction between compounds and targets, we created a “herb-disease-gene-target” network (Fig. 2). Our findings revealed that 3 compounds in THSWD were strongly associated with DVT: kaempferol (degree = 96; C4), beta-sitosterol (degree = 104; C3), and quercetin (degree = 124; HH19).

### 3.3. PPI network construction and analysis

**Figure 3.**
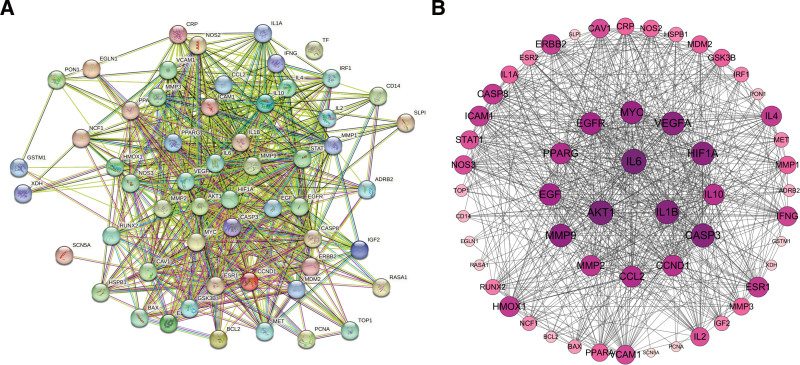
(A) PPI network, (B) PPI network diagram processed by Cytoscape. Empty nodes represent proteins of unknown 3D structures; filled nodes represent some 3D structures that are known or predicted. Edges represent protein-protein associations: the light blue edges represent from curated databases; the fuchsia edges represent experimentally determined; the green edges represent gene neighborhood; the red edges represent gene fusions; the dark blue edges represent gene co-occurrence; the light green edges represent text mining; the black edges represent co-expression; the light purple edges represent protein homology. The color of the target point changes gradually according to the degree value. The higher the degree value, the larger the circle. As the degree value changes, the color changes from light purple to deep purple.

In further analysis, the identified 56 core overlapping targets were uploaded to the STRING database and the species “Homo sapiens” was selected to construct a PPI network (Fig. 3A). The downloaded file was imported into Cytoscape to calculate node degrees and identify key target proteins. This analysis identified potential core targets of THSWD for treating DVT, such as IL1B (degree = 44), IL6 (degree = 46), and AKT1 (degree = 47) (Fig. 3B).

### 3.4. GO and KEGG pathway enrichment analysis

**Figure 4.**
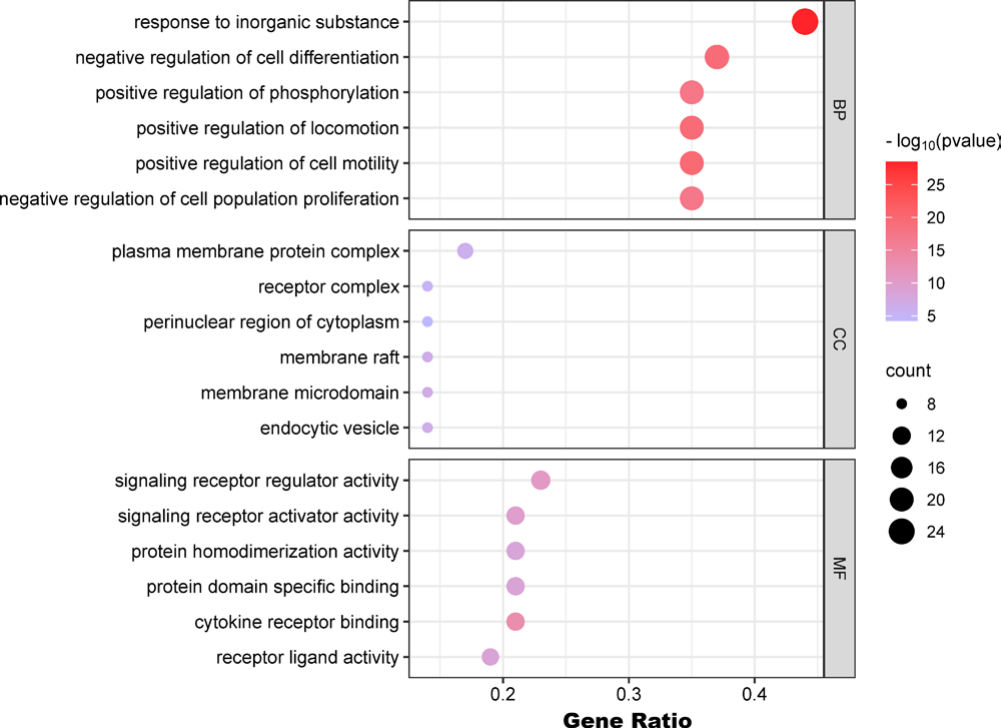
GO analysis of key target genes. The top 5 items of biological function are listed on the vertical axis, including GOMF, GOCC, and GOBP, the horizontal axis in the figure represents the gene ratio. BP = biological process; CC = cell composition; GO = Gene ontology; MF = molecular functions.

**Figure 5.**
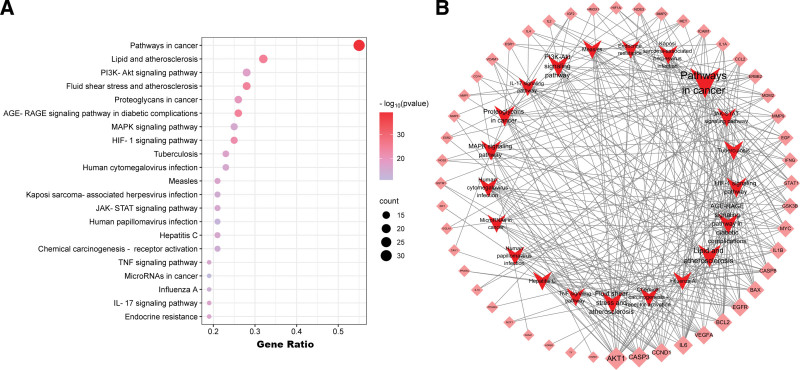
(A) KEGG analysis of key target genes, (B) Network of top 20 pathways. Light red diamond represents gene, and crimson triangle represents pathway. The size of the nodes represents the value of the degree. The horizontal axis in the figure represents the gene ratio. KEGG = Kyoto Encyclopedia of Gene and Genome.

To gain a better understanding of how THSWD can potentially treat DVT, we conducted GO and KEGG enrichment analyses. The top 5 results of the GO analysis are shown in Figure 4, which indicate that THSWD primarily affects the response to inorganic substances (BP), positive regulation of phosphorylation (BP), negative regulation of cell differentiation (BP), plasma membrane protein complex (cell composition), and signaling receptor regulator activity (molecular functions). The KEEG analysis revealed several related signaling pathways, such as lipid and atherosclerosis, pathways in cancer, and the PI3K-Akt signaling pathway (Fig. 5A and B).

### 3.5. Molecular docking

**Figure 6.**
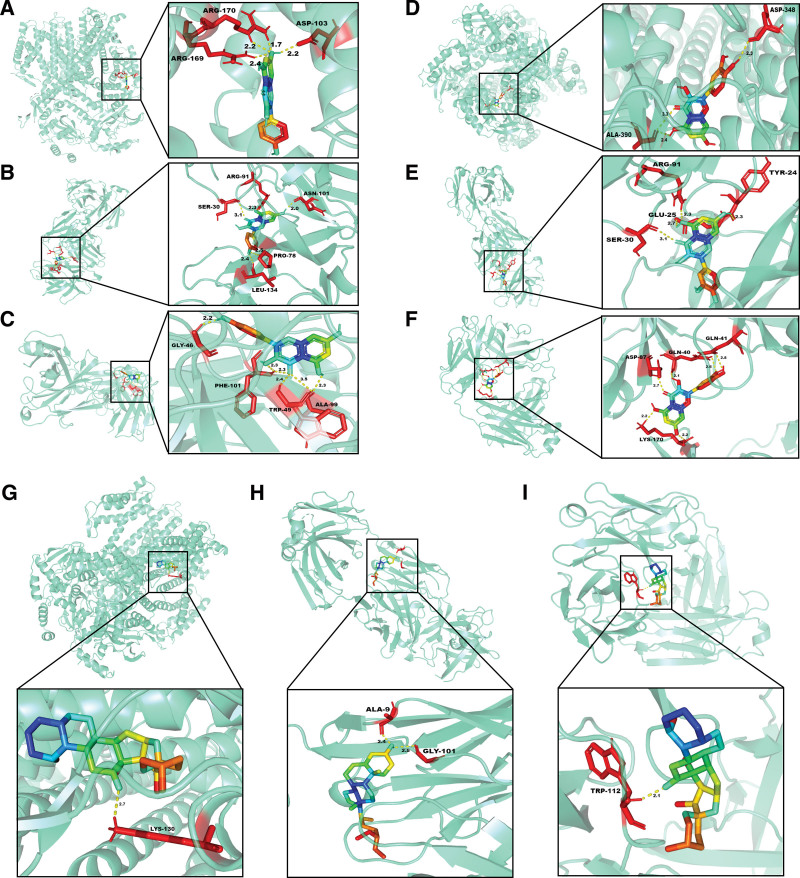
Molecular docking results of main chemical components of THSWD and core proteins in PPI network. (A) kaempferol—AKT1, (B) kaempferol—IL1B, (C) kaempferol—IL6, (D) quercetin—AKT1, (E) quercetin—IL1B, (F) quercetin—IL6, (G) beta-sitosterol—AKT1, (H) beta-sitosterol—IL1B, (I) beta-sitosterol—IL6.

Based on network pharmacology and PPI results, we selected the top 3 compounds: beta-sitosterol, quercetin, and kaempferol. Each compound was individually docked with the top 3 proteins IL1B, IL6, and AKT1. Figure 6 shows the docking image of the receptor-ligand complex that produced the best results.

Our findings suggest that kaempferol can establish hydrogen bonds with the AKT1 protein through ARG-169/170 and ASP-103, as shown in Figure 6A. Additionally, it can form hydrogen bonds with IL1B via ASN-101, LEU-134, PRO-78, ARG-91, and SER-30 (Fig. 6B), and with IL6 via ALA-99, GLY-46, TRP-49, and PHE-101 (Fig. 6C). Quercetin exhibits similar behavior to kaempferol: it forms hydrogen bonds with the AKT1 protein through ASP −348 and ALA −390 (Fig. 6D), interacts with IL1B protein through ARG-91, SER-30, TYR-24, and GLU-25 (Fig. 6E), while interacting with IL6 protein via LYS-170, GLN-40/41, and ASP-87 (Fig. 6F). Similarly beta-sitosterol establishes hydrogen bonds with the AKT1 protein via LYS-130 (Fig. 6G) whereas for IL1B it is done by binding to ALA-9 and GLY-101 (Fig. 6H). Finally beta-sitosterol also binds to IL6 via TRP-112 (Fig. 6I).

According to previous research,^[14]^ a binding energy below -5 kcal/mol indicates strong binding activity between the receptor and ligand. Our molecular docking analysis revealed that beta-sitosterol, quercetin, and kaempferol all displayed high affinity for 3 key targets (IL6, L1B, and AKT1). In fact, their respective binding energies were lower than -5 kcal/mol as shown in Table 1.

**Table 1:**
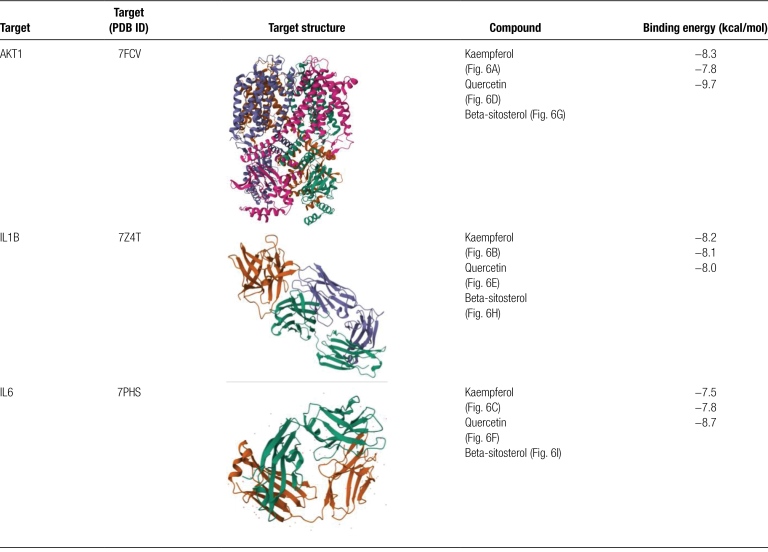
Binding energy of molecular docking.

## 4. Discussion

This study aims to investigate how THSWD treats DVT through network analysis. Our findings demonstrate a significant correlation between various components of THSWD, such as beta-sitosterol, quercetin, kaempferol, and DVT. Previous research has indicated that quercetin and its derivatives possess antifibrinolytic, anticoagulant, and antiplatelet properties.^[35,36]^ Moreover, in mouse models, quercetin has been shown to inhibit thrombosis by inhibiting protein disulfide isomerase (PDI).^[37]^ Both kaempferol and quercetin are flavonoids that regulate the interaction between fibrinogen and thrombin to prevent thrombosis while inhibiting prothrombin activity in vitro and in vivo.^[38]^ Additionally, beta sitosterol derived from plants exhibits unique antithrombotic activity by inhibiting k-carrageenan induced tail thrombosis in mice without cytotoxicity.^[39]^ The 3 active ingredients, quercetin, beta-sitosterol, and kaempferol, are remarkably consistent with the effective ingredients of Huangqi Guizhi Wuwu Decoction that we previously researched for treating DVT.^[1]^ This strengthens our belief in the crucial role these ingredients play in traditional Chinese medicine’s treatment of DVT and boosts our confidence for future experimental research.

The analysis of the PPI network suggests that AKT1, IL6, and IL1B are potential key targets for THSWD in treating DVT. We have previously conducted network pharmacology studies on the treatment of deep vein thrombosis (DVT) using Chinese herbal medicine.^[1]^ Surprisingly, our findings align with the results of this study. It is well-known that IL1B and IL6 are biologically active cytokines,^[40–42]^ and their involvement in cellular inflammation has been linked to DVT occurrence.^[43]^ Therefore, based on these previous studies, we believe that various Chinese herbal medicines may exert anti-DVT effects by targeting IL1B and IL6 as mediators. This provides new insights for developing treatment strategies for DVT.

To investigate how THSWD works against DVT, we conducted GO and KEGG analyses. The results of the GO analysis THSWD primarily affects the response to inorganic substances, positive regulation of phosphorylation, negative regulation of cell differentiation, plasma membrane protein complex, and signaling receptor regulator activity. The KEGG analysis shows that THSWD treatment for DVT involves multiple signaling pathways such as Pathways in Cancer, Lipid and Atherosclerosis, and PI3K-Akt signaling pathways. The quercetin has been identified as one of the main components involved in various cancer pathogenesis mechanisms which may explain its enrichment in the cancer pathway. Quercetin, one of the main components selected by us, has been confirmed to be involved in various cancer pathogenesis mechanisms. This may explain why the KEGG analysis results showed enrichment in the cancer pathway.^[44,45]^

Atherosclerosis and venous thrombosis were once believed to have entirely distinct pathogenesis. However, recent research has challenged this notion.^[46]^ In fact, a study published in the New England Journal of Medicine suggests that atherosclerosis may be a contributing factor to the development of venous thrombosis.^[47]^ Specifically, The development of DVT is caused by an imbalance in maladaptation of the immune response and lipid metabolism. This may lead to chronic inflammation of the vascular wall.^[48,49]^ Chronic inflammation in the blood vessel wall can also affect the ability of thrombotic substances to adhere in the peripheral venous system,^[50]^ increasing the risk of DVT.^[51,52]^ The development of chronic inflammation is significantly influenced by important inflammatory mediators like IL1B and IL6.^[53]^ Our PPI analysis results are consistent with these processes (IL1B, IL6), indicating their crucial role in DVT development.

THSWD has been shown to possess various mechanisms, including the inhibition of inflammatory reactions, anti-atherosclerosis properties, improvement of blood rheology, and regulation of related signaling pathways. These mechanisms make it a potential treatment option for preventing and treating myocardial injury.^[54]^ Given its positive therapeutic effect on atherosclerosis and the interrelation between atherosclerosis and DVT, investigating whether THSWD can simultaneously inhibit the progression of these 2 diseases is worthwhile. Our research results align with one of the goals of network pharmacology—drug repositioning.

The PI3K/Akt signaling pathway plays a critical role in numerous physiological and pathological processes, including cell growth, differentiation, and proliferation.^[55]^ Research has shown that the FXII protein can promote the occurrence of deep vein thrombosis (DVT) by inducing an inflammatory response and activating the PI3K/AKT signaling pathway.^[56]^ IL1B and IL6 are important mediators in this inflammatory response. Increasing evidence suggests that an imbalance in the expression of inflammatory cytokines is closely related to DVT formation.^[57]^ This explains why our protein–protein interaction (PPI) network is enriched with AKT1, IL6, and IL1B proteins. These findings not only validate the crucial role of AKT1 in PPI analysis, but also predict future research directions. It is possible that THSWD may regulate the PI3K/Akt pathway by affecting the inflammatory response, thereby slowing down DVT progression.

The results of molecular docking experiments indicate that the core target proteins IL6, L1B, and AKT1 have strong affinity with the main compounds screened from THSWD. This indicates that these 3 small molecule compounds may bind to these proteins under certain specific and suitable conditions and play a role in delaying the progression of DVT.

This study has several limitations. Firstly, the accuracy and timeliness of the database data may require further improvement. Additionally, our analysis may not include unconfirmed or undocumented compounds or targets. Therefore, while beta-sitosterol, quercetin, and kaempferol have been identified as the main components of THSWD against DVT in our screening process, they do not fully represent THSWD. Consequently, further molecular experiments are necessary to validate our findings and will be the focus of our future research.

## 5. Conclusion

In conclusion, our study indicates that IL6, L1B, and AKT1 could be potential targets for treating DVT with THSWD. The active components responsible for this effect may include beta-sitosterol, quercetin, and kaempferol. These compounds may regulate the process of inflammation through the PI3K/Akt pathway, ultimately slowing down the progression of DVT. However, further basic research is required to validate our findings as it forms the next phase of our research.

## Acknowledgements

This work was supported by Luzhou Science and Technology Program (2022-SYF-41). The authors would like to thank all authors of references.

## Author contributions

**Conceptualization:** Qingyan Liu.

**Data curation:** Wei Fan, Qingyan Liu.

**Formal analysis:** Qingyan Liu.

**Funding acquisition:** Jinhui Liu.

**Investigation:** Jinhui Liu.

**Methodology:** Jinhui Liu.

**Project administration:** Jinhui Liu.

**Resources:** Jinhui Liu.

**Software:** Wei Fan, Qingyan Liu.

**Supervision:** Wei Fan.

**Validation:** Wei Fan.

**Writing – original draft:** Wei Fan, Qingyan Liu.

**Writing – review & editing:** Wei Fan.

## Supplementary Material
