# Supplementary material for: Exploring the potential mechanism and molecular targets of Taohong Siwu Decoction against deep vein thrombosis based on network pharmacology and analysis docking
Source: Medicine (Baltimore). 2024 Jan 12;103(2):e36220. doi: 10.1097/MD.0000000000036220 (PMC10783296; doi:10.1097/MD.0000000000036220)
Supplement: Supplementary file 1 [file medi-103-e36220-s001.docx]

Supplementary file 1.Effective compounds of Taohong Siwu Decoction.

| Name | Mol ID | Mol name | OB (%) | DL |
| --- | --- | --- | --- | --- |
| TR1 | MOL001323 | Sitosterol alpha1 | 43.28 | 0.78 |
| TR2 | MOL001328 | 2,3-didehydro GA70 | 63.29 | 0.5 |
| TR3 | MOL001329 | 2,3-didehydro GA77 | 88.08 | 0.53 |
| TR4 | MOL001339 | GA119 | 76.36 | 0.49 |
| TR5 | MOL001340 | GA120 | 84.85 | 0.45 |
| TR6 | MOL001342 | GA121-isolactone | 72.7 | 0.54 |
| TR7 | MOL001343 | GA122 | 64.79 | 0.5 |
| TR8 | MOL001344 | GA122-isolactone | 88.11 | 0.54 |
| TR9 | MOL001348 | gibberellin 17 | 94.64 | 0.49 |
| TR10 | MOL001349 | 4a-formyl-7alpha-hydroxy-1-methyl-8-methylidene-4aalpha,4bbeta-gibbane-1alpha,10beta-dicarboxylic acid | 88.6 | 0.46 |
| TR11 | MOL001350 | GA30 | 61.72 | 0.54 |
| TR12 | MOL001351 | Gibberellin A44 | 101.61 | 0.54 |
| TR13 | MOL001352 | GA54 | 64.21 | 0.53 |
| TR14 | MOL001353 | GA60 | 93.17 | 0.53 |
| TR15 | MOL001355 | GA63 | 65.54 | 0.54 |
| TR16 | MOL001358 | gibberellin 7 | 73.8 | 0.5 |
| TR17 | MOL001360 | GA77 | 87.89 | 0.53 |
| TR18 | MOL001361 | GA87 | 68.85 | 0.57 |
| TR19 | MOL001368 | 3-O-p-coumaroylquinic acid | 37.63 | 0.29 |
| TR20 | MOL001371 | Populoside_qt | 108.89 | 0.2 |
| TR21 | MOL000296 | hederagenin | 36.91 | 0.75 |
| TR22 | MOL000493 | campesterol | 37.58 | 0.71 |
| HH1 | MOL001771 | poriferast-5-en-3beta-ol | 36.91 | 0.75 |
| HH2 | MOL002680 | Flavoxanthin | 60.41 | 0.56 |
| HH3 | MOL002694 | 4-[(E)-4-(3,5-dimethoxy-4-oxo-1-cyclohexa-2,5-dienylidene)but-2-enylidene]-2,6-dimethoxycyclohexa-2,5-dien-1-one | 48.47 | 0.36 |
| HH4 | MOL002695 | lignan | 43.32 | 0.65 |
| HH5 | MOL002698 | lupeol-palmitate | 33.98 | 0.32 |
| HH6 | MOL002706 | Phytoene | 39.56 | 0.5 |
| HH7 | MOL002707 | phytofluene | 43.18 | 0.5 |
| HH8 | MOL002710 | Pyrethrin II | 48.36 | 0.35 |
| HH9 | MOL002712 | 6-Hydroxykaempferol | 62.13 | 0.27 |
| HH10 | MOL002714 | baicalein | 33.52 | 0.21 |
| HH11 | MOL002717 | qt_carthamone | 51.03 | 0.2 |
| HH12 | MOL002719 | 6-Hydroxynaringenin | 33.23 | 0.24 |
| HH13 | MOL002721 | quercetagetin | 45.01 | 0.31 |
| HH14 | MOL002757 | 7,8-dimethyl-1H-pyrimido[5,6-g]quinoxaline-2,4-dione | 45.75 | 0.19 |
| HH15 | MOL002773 | beta-carotene | 37.18 | 0.58 |
| HH16 | MOL002776 | Baicalin | 40.12 | 0.75 |
| HH17 | MOL000006 | luteolin | 36.16 | 0.25 |
| HH18 | MOL000953 | CLR | 37.87 | 0.68 |
| HH19 | MOL000098 | quercetin | 46.43 | 0.28 |
| BS1 | MOL001910 | 11alpha,12alpha-epoxy-3beta-23-dihydroxy-30-norolean-20-en-28,12beta-olide | 64.77 | 0.38 |
| BS2 | MOL001918 | paeoniflorgenone | 87.59 | 0.37 |
| BS3 | MOL001919 | (3S,5R,8R,9R,10S,14S)-3,17-dihydroxy-4,4,8,10,14-pentamethyl-2,3,5,6,7,9-hexahydro-1H-cyclopenta[a]phenanthrene-15,16-dione | 43.56 | 0.53 |
| BS4 | MOL001921 | Lactiflorin | 49.12 | 0.8 |
| BS5 | MOL001924 | paeoniflorin | 53.87 | 0.79 |
| BS6 | MOL001925 | paeoniflorin_qt | 68.18 | 0.4 |
| BS7 | MOL001928 | albiflorin_qt | 66.64 | 0.33 |
| BS8 | MOL001930 | benzoyl paeoniflorin | 31.27 | 0.75 |
| BS9 | MOL000211 | Mairin | 55.38 | 0.78 |
| BS10 | MOL000492 | (+)-catechin | 54.83 | 0.24 |
| CX1 | MOL001494 | Mandenol | 42 | 0.19 |
| CX2 | MOL002135 | Myricanone | 40.6 | 0.51 |
| CX3 | MOL002140 | Perlolyrine | 65.95 | 0.27 |
| CX4 | MOL002151 | senkyunone | 47.66 | 0.24 |
| CX5 | MOL002157 | wallichilide | 42.31 | 0.71 |
| CX6 | MOL000433 | FA | 68.96 | 0.71 |
| C1 | MOL000359 | sitosterol | 36.91 | 0.75 |
| C2 | MOL000449 | Stigmasterol | 43.83 | 0.76 |
| C3 | MOL000358 | beta-sitosterol | 36.91 | 0.75 |
| C4 | MOL000422 | kaempferol | 41.88 | 0.24 |

Note：(Mol) Molecular, (OB ) Oral bioavailability, (DL) Drug-likeness,(TR) Tao Ren, (HH) Hong Hua, (DG) Dang Gui, (CX) Chuan Xiong, (BS) Bai Shao, (DH) Di Huang, (C1)Sitosterol, common components of DH, BS and CX，(C2) Stigmasterol, common components of DH, HH and DG,(C3) Beta-sitosterol, common components of TR, HH, DG and BS, (C4) Kaempferol ,common components of BS and HH.
